# 2-[(4-Chloro­benz­yl)sulfan­yl]-4-(2-methyl­prop­yl)-6-(phenyl­sulfan­yl)pyrimidine-5-carbonitrile

**DOI:** 10.1107/S1600536812025810

**Published:** 2012-06-13

**Authors:** Ali A. El-Emam, Omar A. Al-Deeb, Abdulghafoor A. Al-Turkistani, Seik Weng Ng, Edward R. T. Tiekink

**Affiliations:** a Department of Pharmaceutical Chemistry, College of Pharmacy, King Saud University, Riyadh 11451, Saudi Arabia; b Department of Chemistry, University of Malaya, 50603 Kuala Lumpur, Malaysia; c Chemistry Department, Faculty of Science, King Abdulaziz University, PO Box 80203 Jeddah, Saudi Arabia

## Abstract

In the title compound, C_22_H_20_ClN_3_S_2_, the *S*-bound benzene rings are inclined [dihedral angles = 78.13 (10) and 36.70 (9)°] with respect to the pyrimidine ring. The methyl­propyl group occupies a position normal to the pyrimidine ring [N—C—C—C torsion angle = 92.3 (2)°]. In the crystal, supra­molecular layers are formed in the *bc* plane, being consolidated by C—H⋯π and π—π inter­actions, the latter between the pyrimidine and *S*-bound benzene rings [inter-centroid distance = 3.7683 (12) Å].

## Related literature

For the chemotherapeutic activity of pyrimidine derivatives, see: Al-Abdullah *et al.* (2011 ▶); Brunelle *et al.* (2007 ▶); Ding *et al.* (2006 ▶); Al-Safarjalani *et al.* (2005 ▶). For a related pyrimidine structure, see: El-Emam *et al.* (2011 ▶).
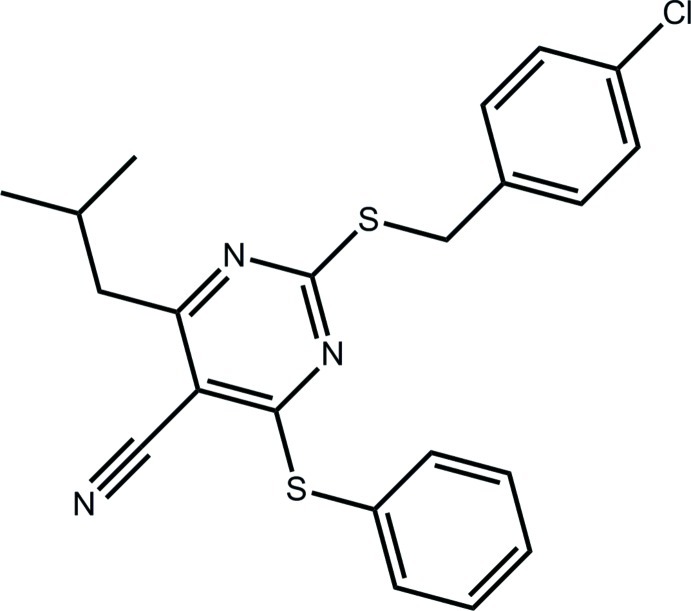


## Experimental

### 

#### Crystal data

C_22_H_20_ClN_3_S_2_
*M* *_r_* = 425.98
Monoclinic, 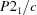

*a* = 13.7771 (2) Å
*b* = 8.4961 (1) Å
*c* = 18.5878 (2) Å
β = 97.559 (1)°
*V* = 2156.82 (5) Å^3^
*Z* = 4
Cu *K*α radiation
μ = 3.47 mm^−1^
*T* = 294 K
0.35 × 0.30 × 0.25 mm

#### Data collection

Agilent SuperNova Dual diffractometer with Atlas detector
Absorption correction: multi-scan (*CrysAlis PRO*; Agilent, 2012 ▶) *T*_min_ = 0.611, *T*_max_ = 1.000
15819 measured reflections
4512 independent reflections
4113 reflections with *I* > 2σ(*I*)
*R*_int_ = 0.018

#### Refinement

*R*[*F* ^2^ > 2σ(*F* ^2^)] = 0.041
*wR*(*F* ^2^) = 0.117
*S* = 1.04
4512 reflections
254 parameters
H-atom parameters constrained
Δρ_max_ = 0.41 e Å^−3^
Δρ_min_ = −0.46 e Å^−3^

### 

Data collection: *CrysAlis PRO* (Agilent, 2012 ▶); cell refinement: *CrysAlis PRO*; data reduction: *CrysAlis PRO*; program(s) used to solve structure: *SHELXS97* (Sheldrick, 2008 ▶); program(s) used to refine structure: *SHELXL97* (Sheldrick, 2008 ▶); molecular graphics: *ORTEP-3 for Windows* (Farrugia, 1997 ▶) and *DIAMOND* (Brandenburg, 2006 ▶); software used to prepare material for publication: *publCIF* (Westrip, 2010 ▶).

## Supplementary Material

Crystal structure: contains datablock(s) general, I. DOI: 10.1107/S1600536812025810/pv2554sup1.cif

Structure factors: contains datablock(s) I. DOI: 10.1107/S1600536812025810/pv2554Isup2.hkl

Supplementary material file. DOI: 10.1107/S1600536812025810/pv2554Isup3.cml

Additional supplementary materials:  crystallographic information; 3D view; checkCIF report

## Figures and Tables

**Table 1:** Hydrogen-bond geometry (Å, °) *Cg*1 is the centroid of the C17–C22 ring.

*D*—H⋯*A*	*D*—H	H⋯*A*	*D*⋯*A*	*D*—H⋯*A*
C6—H6⋯*Cg*1^i^	0.98	2.92	3.789 (2)	148

Symmetry code: (i) 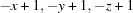.

**Fig. 1.**
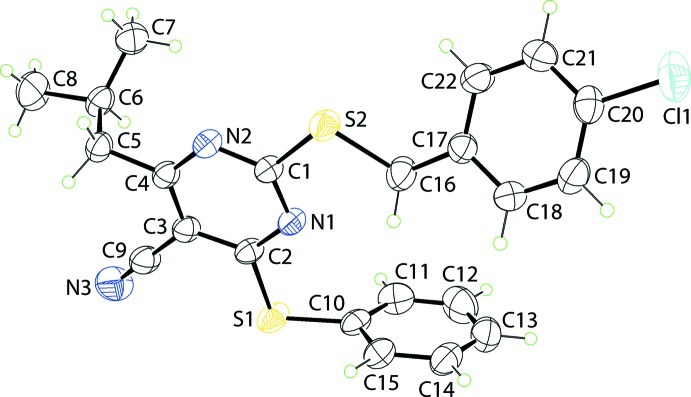
The molecular structure of the title compound showing the atom-labelling scheme and displacement ellipsoids at the 35% probability level.

**Fig. 2.**
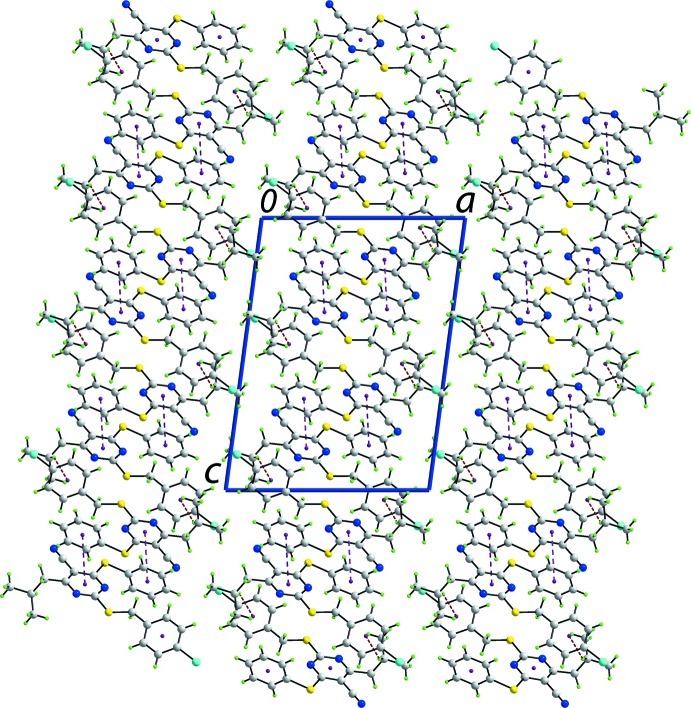
A view in projection down the b axis of the unit-cell contents for the title compound. The C—H···π and π—π interactions are shown as brown and purple dashed lines, respectively.

## supplementary crystallographic information

### Comment

The chemotherapeutic efficacy of pyrimidine derivatives is related to their ability to inhibit vital enzymes responsible for DNA biosynthesis. Thus, several non-nucleoside pyrimidine derivatives exhibit anti-cancer (Al-Safarjalani *et al.*, 2005), anti-viral (Brunelle *et al.*, 2007; Ding *et al.*, 2006) and anti-bacterial activities (Al-Abdullah *et al.*, 2011). In continuation of our interest in the chemical, pharmacological and structural properties of pyrimidine derivatives (El-Emam *et al.*, 2011), we synthesized the title compound as a potential chemotherapeutic agent.

With respect to the pyrimidine ring in the title molecule (Fig. 1), the S1- and S2-bound benzene rings form dihedral angles of 78.13 (10) and 36.70 (9)°, respectively, indicating orthogonal and splayed orientations, respectively; the dihedral angle between the benzene rings = 69.72 (11)°. The methylpropyl group occupies a position normal to the pyrimidine ring with the N2—C4—C5—C6 torsion angle being 92.3 (2)°.

In the crystal packing, supramolecular layers, consolidated by C—H···π, Table 1, and π—π interactions between the pyrimidine and the S1-bound benzene rings [ring centroid(N1,N2,C1–C4)···centroid(C10–C15) distance = 3.7683 (12) Å, angle of inclination = 5.52 (10)° for symmetry operation: 1 - *x*, -1/2 + *y*, 3/2 - *z*], are formed in the *bc* plane, Fig. 2.

### Experimental

To a solution of 2-(4-chlorobenzylsulfanyl)-6-chloro-4-(2-methylpropyl)pyrimidine-5-carbonitrile (705 mg, 2 mmol) in dry pyridine (3 ml), thiophenol (220 mg, 2 mmol) was added and the mixture was heated under reflux for 6 h. On cooling, the solvent was distilled off *in vacuo* and water (5 ml) was added to the residue. The separated precipitate was filtered, washed with cold water, dried and crystallized from ethanol to yield 724 mg (85%) of the title compound as colourless crystals. *M*.pt: 394–396 K. Crystals for the X-ray analysis were obtained by slow evaporation of a solution of the title compound in CHCl_3_:EtOH (1:1, 5 ml) held at room temperature.

### Refinement

Carbon-bound H-atoms were placed in calculated positions [C—H = 0.93 to 0.98 Å, *U*_iso_(H) = 1.2–1.5*U*_eq_(C)] and were included in the refinement in the riding model approximation.

### Crystal data

**Table:**

C_22_H_20_ClN_3_S_2_	*F*(000) = 888
*M**_r_* = 425.98	*D*_x_ = 1.312 Mg m^−^^3^
Monoclinic, *P*2_1_/*c*	Cu *K*α radiation, λ = 1.54184 Å
Hall symbol: -P 2ybc	Cell parameters from 8707 reflections
*a* = 13.7771 (2) Å	θ = 3.8–76.4°
*b* = 8.4961 (1) Å	µ = 3.47 mm^−^^1^
*c* = 18.5878 (2) Å	*T* = 294 K
β = 97.559 (1)°	Prism, colourless
*V* = 2156.82 (5) Å^3^	0.35 × 0.30 × 0.25 mm
*Z* = 4	

### Data collection

**Table:**

Agilent SuperNova Dual diffractometer with Atlas detector	4512 independent reflections
Radiation source: SuperNova (Cu) X-ray Source	4113 reflections with *I* > 2σ(*I*)
Mirror monochromator	*R*_int_ = 0.018
Detector resolution: 10.4041 pixels mm^-1^	θ_max_ = 76.6°, θ_min_ = 4.8°
ω scan	*h* = −16→17
Absorption correction: multi-scan (*CrysAlis PRO*; Agilent, 2012)	*k* = −10→10
*T*_min_ = 0.611, *T*_max_ = 1.000	*l* = −23→22
15819 measured reflections	

### Refinement

**Table:**

Refinement on *F*^2^	Secondary atom site location: difference Fourier map
Least-squares matrix: full	Hydrogen site location: inferred from neighbouring sites
*R*[*F*^2^ > 2σ(*F*^2^)] = 0.041	H-atom parameters constrained
*wR*(*F*^2^) = 0.117	*w* = 1/[σ^2^(*F*_o_^2^) + (0.0576*P*)^2^ + 0.7026*P*] where *P* = (*F*_o_^2^ + 2*F*_c_^2^)/3
*S* = 1.04	(Δ/σ)_max_ < 0.001
4512 reflections	Δρ_max_ = 0.41 e Å^−^^3^
254 parameters	Δρ_min_ = −0.46 e Å^−^^3^
0 restraints	Extinction correction: *SHELXL*, Fc^*^=kFc[1+0.001xFc^2^λ^3^/sin(2θ)]^-1/4^
Primary atom site location: structure-invariant direct methods	Extinction coefficient: 0.0033 (3)

### Special details

**Table:**

Geometry. All s.u.'s (except the s.u. in the dihedral angle between two l.s. planes) are estimated using the full covariance matrix. The cell s.u.'s are taken into account individually in the estimation of s.u.'s in distances, angles and torsion angles; correlations between s.u.'s in cell parameters are only used when they are defined by crystal symmetry. An approximate (isotropic) treatment of cell s.u.'s is used for estimating s.u.'s involving l.s. planes.
Refinement. Refinement of *F*^2^ against ALL reflections. The weighted *R*-factor *wR* and goodness of fit *S* are based on *F*^2^, conventional *R*-factors *R* are based on *F*, with *F* set to zero for negative *F*^2^. The threshold expression of *F*^2^ > σ(*F*^2^) is used only for calculating *R*-factors(gt) *etc*. and is not relevant to the choice of reflections for refinement. *R*-factors based on *F*^2^ are statistically about twice as large as those based on *F*, and *R*- factors based on ALL data will be even larger.

### Fractional atomic coordinates and isotropic or equivalent isotropic displacement parameters (Å^2^)

**Table:**

	*x*	*y*	*z*	*U*_iso_*/*U*_eq_	
S1	0.54678 (4)	0.70944 (6)	0.73269 (3)	0.06701 (18)	
S2	0.49600 (3)	0.22299 (6)	0.55116 (3)	0.06114 (16)	
Cl1	0.03279 (4)	0.03665 (9)	0.36870 (4)	0.0973 (2)	
N1	0.53172 (10)	0.46391 (16)	0.64164 (8)	0.0477 (3)	
N2	0.66645 (10)	0.30012 (16)	0.61951 (8)	0.0505 (3)	
N3	0.80681 (16)	0.6954 (3)	0.78507 (12)	0.0912 (7)	
C1	0.57237 (12)	0.34328 (19)	0.61056 (9)	0.0466 (3)	
C2	0.59250 (12)	0.55031 (19)	0.68709 (9)	0.0474 (4)	
C3	0.69249 (12)	0.5177 (2)	0.70000 (9)	0.0483 (4)	
C4	0.72740 (11)	0.3888 (2)	0.66431 (9)	0.0466 (4)	
C5	0.83447 (12)	0.3509 (2)	0.67331 (10)	0.0541 (4)	
H5A	0.8431	0.2392	0.6652	0.065*	
H5B	0.8631	0.3749	0.7226	0.065*	
C6	0.88789 (13)	0.4457 (2)	0.61966 (11)	0.0578 (4)	
H6	0.8652	0.5549	0.6198	0.069*	
C7	0.86479 (17)	0.3820 (3)	0.54342 (12)	0.0774 (6)	
H7A	0.8969	0.4452	0.5109	0.116*	
H7B	0.7953	0.3850	0.5289	0.116*	
H7C	0.8874	0.2753	0.5421	0.116*	
C8	0.99730 (16)	0.4440 (4)	0.64422 (16)	0.0962 (9)	
H8A	1.0300	0.5050	0.6112	0.144*	
H8B	1.0208	0.3375	0.6450	0.144*	
H8C	1.0104	0.4882	0.6920	0.144*	
C9	0.75677 (14)	0.6152 (3)	0.74776 (11)	0.0616 (5)	
C10	0.42250 (13)	0.7160 (2)	0.69300 (10)	0.0532 (4)	
C11	0.39312 (19)	0.8248 (3)	0.63982 (13)	0.0737 (6)	
H11	0.4385	0.8918	0.6229	0.088*	
C12	0.2950 (2)	0.8330 (3)	0.61170 (15)	0.0912 (8)	
H12	0.2744	0.9069	0.5760	0.109*	
C13	0.22799 (19)	0.7338 (3)	0.63589 (14)	0.0811 (7)	
H13	0.1623	0.7404	0.6166	0.097*	
C14	0.25756 (15)	0.6249 (3)	0.68832 (13)	0.0698 (5)	
H14	0.2122	0.5564	0.7042	0.084*	
C15	0.35460 (14)	0.6169 (2)	0.71770 (11)	0.0592 (4)	
H15	0.3744	0.5445	0.7542	0.071*	
C16	0.37985 (15)	0.3241 (3)	0.55162 (13)	0.0707 (6)	
H16A	0.3653	0.3319	0.6011	0.085*	
H16B	0.3852	0.4301	0.5331	0.085*	
C17	0.29733 (13)	0.2398 (2)	0.50618 (10)	0.0530 (4)	
C18	0.20723 (15)	0.2336 (3)	0.53091 (11)	0.0636 (5)	
H18	0.2008	0.2733	0.5767	0.076*	
C19	0.12653 (15)	0.1699 (3)	0.48935 (12)	0.0672 (5)	
H19	0.0663	0.1673	0.5068	0.081*	
C20	0.13592 (14)	0.1106 (2)	0.42242 (11)	0.0604 (5)	
C21	0.22472 (16)	0.1110 (3)	0.39687 (11)	0.0685 (5)	
H21	0.2309	0.0679	0.3517	0.082*	
C22	0.30503 (15)	0.1760 (3)	0.43874 (11)	0.0636 (5)	
H22	0.3653	0.1769	0.4213	0.076*	

### Atomic displacement parameters (Å^2^)

**Table:**

	*U*^11^	*U*^22^	*U*^33^	*U*^12^	*U*^13^	*U*^23^
S1	0.0521 (3)	0.0639 (3)	0.0867 (4)	−0.0022 (2)	0.0151 (2)	−0.0328 (2)
S2	0.0492 (3)	0.0598 (3)	0.0739 (3)	0.00163 (18)	0.0062 (2)	−0.0236 (2)
Cl1	0.0661 (3)	0.0886 (4)	0.1270 (6)	0.0010 (3)	−0.0249 (3)	−0.0215 (4)
N1	0.0413 (7)	0.0467 (7)	0.0565 (8)	−0.0006 (5)	0.0117 (6)	−0.0069 (6)
N2	0.0446 (7)	0.0483 (7)	0.0603 (8)	0.0020 (6)	0.0134 (6)	−0.0042 (6)
N3	0.0757 (13)	0.1114 (17)	0.0859 (13)	−0.0333 (12)	0.0085 (10)	−0.0302 (12)
C1	0.0442 (8)	0.0445 (8)	0.0527 (8)	−0.0004 (6)	0.0123 (6)	−0.0034 (7)
C2	0.0440 (8)	0.0463 (8)	0.0545 (9)	−0.0042 (6)	0.0163 (7)	−0.0049 (7)
C3	0.0430 (8)	0.0529 (9)	0.0506 (8)	−0.0079 (7)	0.0123 (6)	−0.0027 (7)
C4	0.0413 (8)	0.0495 (8)	0.0510 (8)	−0.0007 (6)	0.0133 (6)	0.0049 (7)
C5	0.0421 (8)	0.0611 (10)	0.0600 (10)	0.0032 (7)	0.0099 (7)	0.0072 (8)
C6	0.0439 (9)	0.0626 (10)	0.0695 (11)	0.0016 (8)	0.0172 (8)	0.0068 (9)
C7	0.0677 (13)	0.1020 (18)	0.0650 (12)	0.0075 (12)	0.0184 (10)	0.0083 (12)
C8	0.0482 (11)	0.144 (3)	0.0971 (18)	−0.0158 (14)	0.0143 (11)	0.0087 (18)
C9	0.0500 (9)	0.0742 (12)	0.0629 (11)	−0.0112 (9)	0.0153 (8)	−0.0114 (10)
C10	0.0537 (9)	0.0465 (9)	0.0624 (10)	0.0039 (7)	0.0183 (8)	−0.0125 (7)
C11	0.0877 (15)	0.0614 (12)	0.0753 (13)	−0.0019 (11)	0.0226 (12)	0.0039 (10)
C12	0.110 (2)	0.0811 (16)	0.0783 (15)	0.0204 (15)	−0.0029 (14)	0.0126 (13)
C13	0.0647 (13)	0.0928 (17)	0.0828 (15)	0.0155 (12)	−0.0022 (11)	−0.0145 (13)
C14	0.0537 (11)	0.0739 (13)	0.0844 (14)	−0.0014 (9)	0.0186 (10)	−0.0115 (11)
C15	0.0572 (10)	0.0526 (10)	0.0701 (11)	0.0050 (8)	0.0168 (9)	0.0003 (8)
C16	0.0530 (10)	0.0725 (13)	0.0839 (14)	0.0089 (9)	−0.0012 (9)	−0.0261 (11)
C17	0.0493 (9)	0.0534 (9)	0.0558 (9)	0.0030 (7)	0.0048 (7)	−0.0034 (8)
C18	0.0588 (11)	0.0810 (13)	0.0525 (10)	0.0087 (10)	0.0126 (8)	−0.0025 (9)
C19	0.0480 (10)	0.0773 (13)	0.0777 (13)	0.0024 (9)	0.0139 (9)	0.0035 (11)
C20	0.0525 (10)	0.0521 (10)	0.0736 (12)	0.0029 (8)	−0.0033 (8)	0.0005 (9)
C21	0.0693 (12)	0.0758 (13)	0.0603 (11)	−0.0030 (10)	0.0081 (9)	−0.0163 (10)
C22	0.0552 (10)	0.0750 (12)	0.0633 (11)	−0.0053 (9)	0.0177 (8)	−0.0113 (10)

### Geometric parameters (Å, º)

**Table:**

S1—C2	1.7559 (16)	C8—H8C	0.9600
S1—C10	1.774 (2)	C10—C11	1.375 (3)
S2—C1	1.7508 (17)	C10—C15	1.382 (3)
S2—C16	1.817 (2)	C11—C12	1.386 (4)
Cl1—C20	1.743 (2)	C11—H11	0.9300
N1—C2	1.330 (2)	C12—C13	1.369 (4)
N1—C1	1.335 (2)	C12—H12	0.9300
N2—C4	1.335 (2)	C13—C14	1.366 (4)
N2—C1	1.336 (2)	C13—H13	0.9300
N3—C9	1.138 (3)	C14—C15	1.378 (3)
C2—C3	1.395 (2)	C14—H14	0.9300
C3—C4	1.398 (2)	C15—H15	0.9300
C3—C9	1.432 (2)	C16—C17	1.505 (3)
C4—C5	1.498 (2)	C16—H16A	0.9700
C5—C6	1.542 (2)	C16—H16B	0.9700
C5—H5A	0.9700	C17—C18	1.380 (3)
C5—H5B	0.9700	C17—C22	1.382 (3)
C6—C7	1.511 (3)	C18—C19	1.378 (3)
C6—C8	1.516 (3)	C18—H18	0.9300
C6—H6	0.9800	C19—C20	1.364 (3)
C7—H7A	0.9600	C19—H19	0.9300
C7—H7B	0.9600	C20—C21	1.369 (3)
C7—H7C	0.9600	C21—C22	1.381 (3)
C8—H8A	0.9600	C21—H21	0.9300
C8—H8B	0.9600	C22—H22	0.9300
			
C2—S1—C10	102.20 (8)	C11—C10—S1	119.80 (16)
C1—S2—C16	100.23 (9)	C15—C10—S1	120.09 (15)
C2—N1—C1	115.71 (14)	C10—C11—C12	119.1 (2)
C4—N2—C1	116.30 (14)	C10—C11—H11	120.5
N1—C1—N2	127.73 (15)	C12—C11—H11	120.5
N1—C1—S2	118.00 (12)	C13—C12—C11	120.8 (2)
N2—C1—S2	114.27 (12)	C13—C12—H12	119.6
N1—C2—C3	121.65 (15)	C11—C12—H12	119.6
N1—C2—S1	119.73 (12)	C14—C13—C12	120.0 (2)
C3—C2—S1	118.62 (13)	C14—C13—H13	120.0
C2—C3—C4	117.89 (15)	C12—C13—H13	120.0
C2—C3—C9	120.51 (16)	C13—C14—C15	120.0 (2)
C4—C3—C9	121.59 (16)	C13—C14—H14	120.0
N2—C4—C3	120.71 (15)	C15—C14—H14	120.0
N2—C4—C5	118.54 (15)	C14—C15—C10	120.1 (2)
C3—C4—C5	120.72 (16)	C14—C15—H15	119.9
C4—C5—C6	111.24 (15)	C10—C15—H15	119.9
C4—C5—H5A	109.4	C17—C16—S2	111.90 (14)
C6—C5—H5A	109.4	C17—C16—H16A	109.2
C4—C5—H5B	109.4	S2—C16—H16A	109.2
C6—C5—H5B	109.4	C17—C16—H16B	109.2
H5A—C5—H5B	108.0	S2—C16—H16B	109.2
C7—C6—C8	110.93 (18)	H16A—C16—H16B	107.9
C7—C6—C5	111.11 (17)	C18—C17—C22	117.82 (18)
C8—C6—C5	109.91 (18)	C18—C17—C16	118.50 (17)
C7—C6—H6	108.3	C22—C17—C16	123.56 (18)
C8—C6—H6	108.3	C19—C18—C17	121.52 (18)
C5—C6—H6	108.3	C19—C18—H18	119.2
C6—C7—H7A	109.5	C17—C18—H18	119.2
C6—C7—H7B	109.5	C20—C19—C18	119.33 (19)
H7A—C7—H7B	109.5	C20—C19—H19	120.3
C6—C7—H7C	109.5	C18—C19—H19	120.3
H7A—C7—H7C	109.5	C19—C20—C21	120.78 (19)
H7B—C7—H7C	109.5	C19—C20—Cl1	119.19 (16)
C6—C8—H8A	109.5	C21—C20—Cl1	120.02 (16)
C6—C8—H8B	109.5	C20—C21—C22	119.44 (19)
H8A—C8—H8B	109.5	C20—C21—H21	120.3
C6—C8—H8C	109.5	C22—C21—H21	120.3
H8A—C8—H8C	109.5	C21—C22—C17	121.07 (18)
H8B—C8—H8C	109.5	C21—C22—H22	119.5
N3—C9—C3	178.5 (3)	C17—C22—H22	119.5
C11—C10—C15	120.0 (2)		
			
C2—N1—C1—N2	−0.4 (3)	C2—S1—C10—C11	−100.58 (16)
C2—N1—C1—S2	179.05 (12)	C2—S1—C10—C15	82.29 (16)
C4—N2—C1—N1	−0.7 (3)	C15—C10—C11—C12	0.0 (3)
C4—N2—C1—S2	179.89 (12)	S1—C10—C11—C12	−177.11 (19)
C16—S2—C1—N1	2.66 (17)	C10—C11—C12—C13	−0.5 (4)
C16—S2—C1—N2	−177.85 (15)	C11—C12—C13—C14	0.0 (4)
C1—N1—C2—C3	1.2 (2)	C12—C13—C14—C15	1.1 (4)
C1—N1—C2—S1	−178.59 (12)	C13—C14—C15—C10	−1.6 (3)
C10—S1—C2—N1	−5.74 (16)	C11—C10—C15—C14	1.0 (3)
C10—S1—C2—C3	174.49 (14)	S1—C10—C15—C14	178.16 (15)
N1—C2—C3—C4	−0.9 (2)	C1—S2—C16—C17	−177.46 (16)
S1—C2—C3—C4	178.83 (12)	S2—C16—C17—C18	141.33 (18)
N1—C2—C3—C9	178.32 (17)	S2—C16—C17—C22	−42.7 (3)
S1—C2—C3—C9	−1.9 (2)	C22—C17—C18—C19	−1.7 (3)
C1—N2—C4—C3	0.9 (2)	C16—C17—C18—C19	174.6 (2)
C1—N2—C4—C5	−176.83 (15)	C17—C18—C19—C20	0.4 (3)
C2—C3—C4—N2	−0.2 (2)	C18—C19—C20—C21	1.3 (3)
C9—C3—C4—N2	−179.41 (16)	C18—C19—C20—Cl1	−177.69 (17)
C2—C3—C4—C5	177.52 (15)	C19—C20—C21—C22	−1.7 (3)
C9—C3—C4—C5	−1.7 (3)	Cl1—C20—C21—C22	177.29 (18)
N2—C4—C5—C6	92.3 (2)	C20—C21—C22—C17	0.4 (3)
C3—C4—C5—C6	−85.4 (2)	C18—C17—C22—C21	1.2 (3)
C4—C5—C6—C7	−73.9 (2)	C16—C17—C22—C21	−174.8 (2)
C4—C5—C6—C8	162.9 (2)		

### Hydrogen-bond geometry (Å, º)

Cg1 is the centroid of the C17–C22 ring.

**Table:**

*D*—H···*A*	*D*—H	H···*A*	*D*···*A*	*D*—H···*A*
C6—H6···*Cg*1^i^	0.98	2.92	3.789 (2)	148

Symmetry code: (i) −*x*+1, −*y*+1, −*z*+1.
